# Listeria monocytogenes Meningitis in an Intensive Care Unit

**DOI:** 10.7759/cureus.100894

**Published:** 2026-01-06

**Authors:** Ana Sofia Alves, Joana Fernandes, Maria João Pinto, Nelson Barros

**Affiliations:** 1 Intensive Care Unit, Centro Hospitalar de Trás-os-Montes e Alto Douro, Vila Real, PRT

**Keywords:** community-acquired bacterial meningitis, elderly, fever, focal neurological deficits, immunocompromised adults, intensive care unit, listeria monocytogenes

## Abstract

Community-acquired bacterial meningitis remains one of the most serious and life-threatening infectious diseases, with *Listeria monocytogenes* representing the third most common causative agent. *Listeria* meningitis occurs more frequently in immunocompromised and older adults and often requires intensive care unit (ICU) admission, depending on disease severity. We conducted a descriptive, retrospective study in an ICU that included adult patients admitted with community-acquired *L. monocytogenes* meningitis. Clinical features, risk factors, and management of these patients were analyzed. Over nine years, six patients were admitted to the ICU. The mean ICU stay was 5.8±4.7 days, and the mean hospital stay was 27.8±22.4 days. ICU severity scores at admission were as follows: Acute Physiology and Chronic Health Evaluation II (APACHE II) 19±5.1, Simplified Acute Physiology Score II (SAPS II) 44.3±11.8, SAPS III 53.3±11.6, and Sequential Organ Failure Assessment (SOFA) 6±3.2. The classic triad of meningitis (fever, neck stiffness, and altered mental status) was present in only two patients (33.3%). Lumbar puncture and blood cultures were performed in all patients. The most relevant risk factors identified were advanced age and alcohol consumption. All patients received the first dose of antibiotics in the emergency department before ICU admission. Despite the associated morbidity, all patients survived, although two experienced neurological sequelae.

## Introduction

Meningitis is an inflammation of the meninges and the subarachnoid space that may produce the classic triad of headache, fever, and neck stiffness [1,2]. Acute meningitis may be caused by multiple infectious pathogens, with bacterial meningitis representing the most severe form [3]. Additionally, it may occur as a manifestation of non-infectious diseases, although this is uncommon.

Meningitis may be classified by etiology as community-acquired, occurring spontaneously, or nosocomial, developing as a complication of invasive procedures or head trauma [1]. Community-acquired bacterial meningitis remains one of the most serious and life-threatening infectious diseases worldwide, despite advances in diagnosis and treatment. Epidemiological data indicate that *Streptococcus pneumoniae* and *Neisseria meningitidis* are the leading causes, followed by *Listeria monocytogenes* as the third most frequent pathogen [3-5].

*L. monocytogenes* is a gram-positive, facultative anaerobic bacillus [4-6]. Meningitis caused by this organism may occur in both immunocompetent and immunocompromised individuals, although it predominantly affects older adults and those with impaired immunity [1,4-6]. Established risk factors include age >60 years, alcoholism, diabetes mellitus, hypertension, smoking, cancer, immunosuppressive therapy, organ transplantation, and other conditions associated with immunosuppression [7-9].

*L. monocytogenes* meningitis is associated with high morbidity and mortality, even when diagnosed early and treated promptly [3,10,11]. Therefore, it should be managed as a medical emergency, often requiring intensive care unit (ICU) admission and adherence to current treatment recommendations from the Infectious Diseases Society of America (IDSA) and the European Society of Clinical Microbiology and Infectious Diseases (ESCMID) [12,13].

Despite its severity and poor prognosis, few studies have evaluated *L. monocytogenes* meningitis in the ICU setting, and most existing reports focus on general disease characteristics in adults [2,4,6-11]. This study aimed to evaluate the clinical features, risk factors, and acute management of adult patients admitted to an ICU with *L. monocytogenes* meningitis and to describe the complications observed in these patients.

## Materials and methods

A descriptive, retrospective study was conducted in the ICU of a regional hospital in Portugal. The study period extended from January 2016 to December 2024. Electronic medical records were reviewed using the SClinic system, the platform utilized at the study center, and the ICU database maintained by ICU leadership. Approval for the study was granted by the head of the Department of Emergency and Intensive Care, who determined that informed consent was not required.

The inclusion criteria were as follows: patients aged >18 years with *L. monocytogenes* meningitis, diagnosed based on clinical symptoms and confirmed by cerebrospinal fluid (CSF) culture and *Listeria* identification. Meningitis was classified as community-acquired if the patient had not been hospitalized in the preceding six months and did not reside in a long-term care facility. Exclusion criteria included positive blood cultures with negative CSF cultures, residence in nursing or long-term care facilities, and hospital admission within the previous six months.

Patients were classified as older adults if they were aged >60 years. Immunocompromised status was defined as the use of immunosuppressive drugs, the presence of malignancy, connective tissue disease, or prior organ transplantation. Risk factors considered relevant for *Listeria* meningitis included alcoholism, diabetes mellitus, hypertension, and active smoking.

ICU severity scores assessed at admission included the Acute Physiology and Chronic Health Evaluation II (APACHE II) [14], Simplified Acute Physiology Score II (SAPS II) [15], SAPS III [16], and the Sequential Organ Failure Assessment (SOFA) [17] score. Patients were evaluated for sepsis and septic shock according to established criteria. Organ dysfunctions were recorded. Respiratory dysfunction included the need for invasive mechanical ventilation and the development of acute respiratory distress syndrome (ARDS), as defined by the Berlin classification.

Symptom duration before admission was categorized as <24 hours, <48 hours, or >72 hours. Clinical characteristics at admission included the presence of the classic triad (headache, fever, and neck stiffness). The Glasgow Coma Scale (GCS) score was recorded at admission. Focal neurological deficits were identified when aphasia, hemiparesis, cranial nerve palsies, and cerebellar dysfunction (e.g., ataxia) were present. Patients who required cranial CT before lumbar puncture were identified.

CSF parameters evaluated included white blood cell count, protein, glucose, and the CSF-to-blood glucose ratio. Laboratory variables included leukocyte count, glucose, sodium, and C-reactive protein (CRP), and blood cultures positive for *L. monocytogenes* were recorded.

The time to initiation of empirical antibiotics, the empirical antibiotic therapy, and the targeted therapeutic regimen were evaluated. Additionally, the use of adjunctive dexamethasone was recorded.

Patients were followed until death or hospital discharge. Before hospital discharge, patients were assessed for neurological sequelae. 

Statistical analysis was performed using Microsoft Excel (Microsoft Corp., Redmond, WA). A descriptive analysis of all variables was conducted. Continuous variables, including age, ICU and hospital length of stay, ICU severity scores, GCS score at presentation, CSF, and laboratory findings, were expressed as mean ± SD. Categorical variables, including male sex, ICU admission within <24 hours, immunocompromised status, risk factors, duration of symptoms before admission, symptoms at presentation, focal neurological deficits, microbiological findings, and outcomes, were presented as percentages. No inferential statistical tests were performed, as this study was purely descriptive.

## Results

Over the nine-year study period, six patients were diagnosed with community-acquired *L. monocytogenes* meningitis, which represents the rarity of this disease. The demographic, clinical, and laboratory characteristics are summarized in Table 1. The mean age was 65.5±7.9 years (range: 58-76), with five patients aged >60 years and one patient aged 58 years. Four patients (66.7%) were male. The average ICU stay was 5.8±4.7 days (range: 2-15), and the mean hospital stay was 27.8±22.4 days. ICU admission occurred within 24 hours of hospital presentation in four patients (66.7%).

**Table 1:** Demographic, clinical, and laboratory characteristics at ICU admission in patients with Listeria monocytogenes meningitis ICU, intensive care unit; APACHE II, Acute Physiology and Chronic Health Evaluation II; SAPS II, Simplified Acute Physiology Score II; SAPS III, Simplified Acute Physiology Score 3; SOFA, Sequential Organ Failure Assessment; CSF, cerebrospinal fluid; CRP: C-reactive protein

Variable	Patients (n=6)
Age (mean years ± SD)	65.5±7.9
Age 50-60 years	1 (58 years)
Age ≥60 years	5 (mean 67 years)
Male sex	4 (66.7%)
Length of ICU stay (mean days ± SD)	5.8±4.7
Length of hospital stay (mean days ± SD)	27.8±22.4
ICU admission <24 hours after hospital presentation	4 (66.7%)
ICU severity scores	
APACHE II (mean ± SD)	19±5.1
SAPS II (mean ± SD)	44.3±11.8
SAPS III (mean ± SD)	53.3±11.6
SOFA at admission (mean ± SD)	6±3.2
Immunosuppression	
Rheumatoid arthritis on immunosuppressive therapy	1 (16.7%)
Risk factors	
Hypertension	3 (50%)
Alcohol consumption	3 (50%)
Smoking	2 (33.3%)
Diabetes mellitus	1 (16.7%)
Duration of symptoms before admission	
<24 hours	3 (50%)
<48 hours	2 (33.3%)
>72 hours	1 (16.7%)
Symptoms at presentation	
Fever >38ºC	6 (100%)
Neck stiffness	4 (66.7%)
Headache	3 (50%)
Vomiting	2 (33.3%)
Seizures	2 (33.3%)
Classic triad	2 (33.3%)
Glasgow Coma Scale score (mean ± SD)	12.7±1.2
Focal neurological deficits	
Any	4 (66.7%)
Aphasia	2 (33.3%)
Hemiparesis	1 (16.7%)
Cranial nerve palsies	1 (16.7%)
Ataxia	1 (16.7%)
Laboratory findings	
CSF findings	
White blood cell count (mean ± SD)	661±539.1 cells/µL
Protein level (mean ± SD)	3.8±1.6 g/L
Glucose (mean ± SD)	21.5±31.5 mg/mL
CSF-to-blood glucose ratio (mean ± SD)	0.14±0.18
Laboratory findings - blood	
White blood cell count (mean ± SD)	13,468±2,261.7 cells/µL
CRP level (mean ± SD)	7.2±3.5 mg/dL
Glucose (mean ± SD)	119.2±34.5 mg/mL
Serum sodium (mean ± SD)	134.3±4.5 mEq/L
Cultures	
CSF culture with *Listeria monocytogenes*	6 (100%)
Blood cultures with *Listeria* monocytogenes	3 (50%)
Outcome	
Survival	6 (100%)
Neurological sequelae	2 (33.3%)
Mortality	0

ICU severity scores at admission were as follows: mean APACHE II, 19±5.1 (range: 16-28); SAPS II, 44.3±11.8 (range: 28-56); SAPS III, 53.3±11.6 (range: 33-68); and mean SOFA score, 6±3.2. Five patients (83.3%) met the criteria for sepsis, and one (16.7%) met the criteria for septic shock. Four patients (66.7%) required vasopressor support for cardiovascular dysfunction, and one patient (16.7%) required renal replacement.

Four patients (66.7%) required invasive mechanical ventilation, with a mean duration of four days. Two patients developed ARDS, one moderate and one severe; the latter required prone positioning for alveolar recruitment. One patient was extubated to noninvasive mechanical ventilation, and one patient (16.7%) required re-intubation two hours after extubation due to stridor and laryngeal edema.

Risk factors included diabetes in one patient (16.7%), hypertension in three (50%), alcohol consumption in three (50%), smoking in two (33.3%), and rheumatoid arthritis under immunosuppressive therapy in one patient (16.7%). Symptom onset was within 24 hours before admission in three patients (50%), within 48 hours in two patients (33.3%), and >72 hours in one patient (16.7%).

Clinical features at presentation included fever >38°C in all patients, neck stiffness in four (66.7%), headache in three (50%), vomiting in two (33.3%), and seizures in two (33.3%). The GCS score at presentation was 12.7±1.2 (range: 11-14), and no patient was in a coma (GCS ≤8). The classic triad of fever, neck stiffness, and altered mental status was present in two patients (33.3%). Focal neurological deficits were observed in four patients (66.7%), including aphasia in two (33.3%), hemiparesis in one (16.7%), ataxia in one (16.7%), and cranial nerve palsy in one (16.7%).

Brain CT was performed before lumbar puncture in five patients (83.3%). *L. monocytogenes* was identified in the CSF of all patients, and blood cultures were positive in three cases (50%).

CSF analysis revealed a mean white blood cell count of 661±539.1 cells/μL (range: 386-1490), protein 3.8±1.6 g/L (range: 2.4-6.7), and glucose 21.5±31.5 mg/dL (range: 1-85). The mean CSF-to-blood glucose ratio was 0.14±0.18 (range: 0.01-0.5). Blood tests showed a mean leukocyte count of 13,468±2,261.7 cells/μL, glucose 119.2±34.5 mg/dL, sodium 134.3±4.5 mEq/L, and CRP 7.2±3.5 mg/dL.

Treatment details are provided in Table 2. All patients received the first dose of antibiotics in the emergency department before ICU admission. Empirical antibiotic regimens included ceftriaxone, ampicillin, and vancomycin in two patients; ceftriaxone and ampicillin in two patients; and ceftriaxone alone in two patients. Targeted therapy for *L. monocytogenes* meningitis consisted of ampicillin alone in four patients (66.7%) and ampicillin plus gentamicin in two patients (33.3%). One patient received adjunctive dexamethasone.

**Table 2:** Treatment of patients with Listeria monocytogenes meningitis ICU, intensive care unit

Variable	Patients (n=6)
Antibiotics before ICU admission	6 (100%)
Empirical antibiotics	
Ceftriaxone + ampicillin + vancomycin	2 (33.3%)
Ceftriaxone + ampicillin	2 (33.3%)
Ceftriaxone	2 (33.3%)
Targeted therapy	
Ampicillin	4 (66.7%)
Ampicillin + gentamicin	2 (33.3%)
Adjunctive dexamethasone	
No	5 (83.3%)
Yes	1 (16.7%)

All patients survived, although two experienced neurological sequelae.

## Discussion

This study provides additional information regarding the epidemiology, clinical presentation, disease severity, and outcomes of community-acquired *L. monocytogenes* meningitis in patients admitted to the ICU. These findings may help improve the management of such patients in ICU settings.

The findings indicated that meningitis due to *L. monocytogenes* is more prevalent among older adults and immunocompromised patients. In this cohort, five patients (83.3%) were aged >60 years, consistent with previous reports on the epidemiology of the disease [2-4,6,8-11]. Age was the most relevant predisposing factor for *L. monocytogenes* meningitis, with only one patient having an autoimmune disease (rheumatoid arthritis) under immunosuppressive therapy. Similar to other series, the disease was more prevalent in males [2,4,6-11].

This study included only patients with *L. monocytogenes* admitted to the ICU, rather than all patients with the disease admitted to the center, which represents a limitation. Comparisons with previous cohorts from the United Kingdom [9] and Lithuania [10] show ICU admission rates of 13% and 82.4%, respectively. In the present series, six patients required ICU admission over nine years. Among the ICU severity scores applied, APACHE II [14] demonstrated the strongest association with morbidity and mortality.

The mean SOFA [17] score at ICU admission was 6±3.2. Five patients (83.3%) met the criteria for sepsis, and one (16.7%) met the criteria for septic shock. These findings underscore the importance of recognizing the potential severity of *L. monocytogenes* meningitis at presentation in the emergency department to ensure appropriate resource allocation. The most prevalent organ dysfunctions were neurological, cardiovascular, and respiratory. Four patients required vasopressor support and invasive mechanical ventilation (66.7%), and one (16.7%) required renal replacement therapy.

Among the four patients requiring mechanical invasive ventilation, the mean duration was four days, and two developed ARDS. ARDS is a clinical syndrome of acute respiratory failure caused by lung inflammation, unrelated to cardiogenic pulmonary edema [18]. Severity was classified according to the Berlin 2012 definition, the standard at the time of data collection [18]. One patient developed moderate ARDS (PaO_2_/FiO_2_ ≤200 mmHg with PEEP ≥5 cm H_2_O), and one developed severe ARDS (PaO_2_/FiO_2_ ≤100 mmHg with PEEP ≥5 cm H_2_O) [18], requiring prone positioning for alveolar recruitment [19]. No unplanned extubations occurred; one patient was extubated to noninvasive mechanical ventilation, and another required re-intubation due to extubation failure.

*L. monocytogenes* meningitis can contribute to ARDS through indirect (extrapulmonary) lung injury mediated by inflammatory cytokines affecting the vascular endothelium [18]. This neurogenic mechanism should be considered during management because of its potential to cause respiratory failure.

The length of ICU stay was 5.8±4.7 days (range: 2-15), and the mean hospital stay was 27.8±22.4 days. These patients often required physical rehabilitation after ICU discharge, and treatment duration was at least 21 days. The time to ICU admission was <24 hours in four patients (66.7%), consistent with findings in other series [9,10].

The clinical presentation of *L. monocytogenes* meningitis was similar to that reported in adults with community-acquired bacterial meningitis. Seizures occurred in two patients (33.3%), a symptom more characteristic of *Listeria* meningitis and higher than reported in other series [4,10]. Focal neurological deficits were observed in four patients (66.7%), including aphasia in two (33.3%), hemiparesis in one (16.7%), ataxia in one (16.7%), and cranial nerve palsy in one (16.7%). All patients had fever >38°C, and two patients (33.3%) presented with the classic triad (fever, neck stiffness, and change in mental status), consistent with previous reports [4,8,10]. Notably, no patient presented with coma (GCS ≤8), even in the presence of seizures or focal neurological deficits.

Symptom onset occurred within 24 hours before admission in most patients and in one case more than 72 hours prior, reflecting the subacute nature of *L. monocytogenes* meningitis [20].

Neuroimaging before lumbar puncture was performed in five patients (83.3%), consistent with indications such as focal neurological deficits, seizures, GCS ≤12, or previous neurological disease [9]. *L. monocytogenes* was identified in the CSF of all patients. Invasive listeriosis, as evidenced by positive blood cultures, was present in three patients (50%), lower than in the Netherlands cohort (73%) [21]. CSF analysis findings were consistent with the literature.

Given the high morbidity and mortality of community-acquired bacterial meningitis, empirical antibiotic therapy should be initiated promptly [20,22], even before the diagnosis is confirmed. In this cohort, all patients received the first antibiotic dose in the emergency department. Empirical therapy should be guided by age, risk factors, and local resistance patterns. In two patients (33.3%), empirical antibiotics were inappropriate. Delayed treatment increases the risk of neurological sequelae, which occurred in two patients, as described in previous studies [7,22]. Targeted therapy followed current IDSA and ESCMID guidelines for bacterial meningitis [12,13].

The role of dexamethasone in *L. monocytogenes* meningitis remains unclear. While randomized controlled trials and meta-analyses support dexamethasone as adjuvant therapy in bacterial meningitis for improved hospital outcomes, a survival benefit has not been established [4,23]. Guidelines recommend discontinuing dexamethasone if *L. monocytogenes* is identified, based on the MONALISA study, which showed increased mortality with adjunctive dexamethasone use in these patients [24]. A prospective study conducted in the Netherlands (2006-2022) evaluated the use of adjunctive dexamethasone in 162 patients with *Listeria* meningitis. The results demonstrated improved outcomes and suggested that dexamethasone should be discontinued when *L. monocytogenes* is identified [23]. In the present study, one patient received adjunctive dexamethasone and had a favorable outcome. 

Despite the morbidity and mortality associated with *L. monocytogenes* meningitis, all patients in this cohort survived [3,8,20,25].

This study has some limitations. First, *L. monocytogenes* meningitis is a rare disease, and our cohort included only six patients, resulting in a small sample size. Second, the study was retrospective and descriptive, based on medical records. Third, genotyping of *L. monocytogenes* was not performed because it was unavailable at our center, unlike in other studies [1,5,17,25]. Finally, only patients with positive CSF for *L. monocytogenes* were included.

## Conclusions

This study describes the characteristics of patients with *L. monocytogenes* meningitis admitted to the ICU, including disease severity and the spectrum of organ dysfunctions observed. Given the limited number of studies and reports on this topic, these findings are relevant for other physicians managing this condition in both emergency department and ICU settings. *L. monocytogenes* is a well-recognized cause of community-acquired bacterial meningitis and should be considered in patients aged >60 years, those with a history of alcohol consumption, or immunocompromised individuals. Empirical antibiotic therapy in such cases should include ampicillin. In this series, two patients did not receive ampicillin initially and subsequently developed neurological sequelae. Invasive listeriosis was identified in three patients, one of whom did not receive ampicillin as part of the initial therapy.

Two patients required invasive mechanical ventilation and developed ARDS, highlighting the need to recognize this syndrome in patients with *L. monocytogenes* meningitis presenting with acute respiratory failure. Further studies are required to clarify the role of adjunctive dexamethasone in the treatment of *Listeria* meningitis and to determine whether it should be discontinued when the pathogen is identified.
